# Enhancing Trauma-Informed Practice: A Quality Improvement Project in Healthcare Settings

**DOI:** 10.1192/bjo.2024.405

**Published:** 2024-08-01

**Authors:** Magd Nojoum

**Affiliations:** Queens Hospital, London, United Kingdom

## Abstract

**Aims:**

This pilot study addresses the implementation of trauma-informed practice within healthcare settings. Given the profound impact of trauma on individuals’ health and well-being, there's an increasing recognition of the importance of integrating trauma-informed care into healthcare systems. This project aims to evaluate and enhance healthcare professionals' understanding, awareness, and confidence in implementing trauma-informed practices through targeted interventions.

**Methods:**

The project initiated with a baseline assessment through surveys among healthcare professionals to gauge their initial understanding, awareness, and confidence levels in applying trauma-informed practices in their work environments (n = 9). Subsequently, a structured teaching session was conducted to provide education and training on trauma-informed care. Post-session, a reassessment survey measured improvements in awareness, understanding, and confidence levels (n = 5).

Following this, a visual aid – a comprehensive poster summarizing key aspects of trauma-informed practice – was created and displayed prominently in healthcare settings. A second cycle of the quality improvement initiative was undertaken, measuring outcomes after the implementation of the poster. Surveys were administered again to evaluate the impact of the visual aid on sustaining and further enhancing healthcare professionals’ adherence to trauma-informed practices (n = 3).

**Results:**

Post-teaching session assessments demonstrated a notable improvement in levels of awareness (44%), and confidence (56%) among healthcare professionals regarding trauma-informed practices, as well as recognition of signs & symptoms of trauma (44%). Subsequent to the poster's introduction, the second cycle of assessments showcased sustained levels of awareness, understanding, and confidence among the participants.

**Conclusion:**

The project underscores the effectiveness of targeted interventions – educational sessions and visual aids – in augmenting healthcare professionals’ understanding, awareness, and confidence in implementing trauma-informed practices. The improvement in these metrics post-interventions emphasizes the value of ongoing education and visual support tools in fostering a trauma-informed approach within healthcare settings. Embedding such practices can significantly impact patient care, fostering a more supportive and empathetic environment for individuals affected by trauma.

